# Comparison of Modern Drinking Water Network Maintenance Methods: Evaluation of Removed Deposits in the Form of Total Suspended Solids (TSS)

**DOI:** 10.3390/ijerph18084311

**Published:** 2021-04-19

**Authors:** Petra Jurek Vidlářová, Silvie Heviánková

**Affiliations:** Department of Environmental Engineering, Faculty of Mining and Geology, VSB-Technical University of Ostrava, 708 00 Ostrava, Czech Republic; silvie.heviankova@vsb.cz

**Keywords:** drinking water distribution system (DWDS), unidirectional flushing, air scouring, Ice Pigging^®^, Comprex^®^, total suspended solids (TSS)

## Abstract

Water pipe sediment removal should be implemented as an integral part of water mains maintenance in order to steadily supply consumers with drinking water of high quality. Considering the number of different water pipe sediment removal methods, the article aims to evaluate the currently used methods to remove water pipe sediment from the pipes of the drinking water distribution system. The evaluation compares the implementation requirements of each method as well as the quality and the quantity of the removed products. The tested methods were unidirectional flushing, Comprex^®^, and Ice Pigging^®^. The results of the comparison are expressed in terms of total suspended solids (TSS) recovery, metals mass concentration and water consumption. Since contamination can settle along the entire surface of the pipeline, it is most appropriate to recalculate the results per unit area of the pipeline. The results point at the following efficiency the Comprex^®^ method was the most efficient in removing TSS, Ice Pigging^®^ was the next and unidirectional flushing removed a negligible amount of TSS compared to the other two methods. The absolute recovery of TSS was 0.12–3.01 g·m^−2^ in unidirectional flushing of plastic pipes, 1.58–8.54 g·m^−2^ in unidirectional flushing of metal pipes, 4.36–47.53 g·m^−2^ in Ice Pigging^®^, and 5.19–69.23 g·m^−2^ in Comprex^®^. The composition of the sediment was strongly influenced by particle origin: Pipe material affected the crystalline phase of the sediment and the water source and the age of the pipe affected the amorphous phase of the sediment. Therefore, it was found that evaluation of efficiency based on the amount of TSS removed is only suitable for sites that meet the same conditions as pipe material, water source and ideally the pipe age. It has further been found that the Comprex^®^ method can be advantageously used in real conditions to clean pipes with insufficient hydraulic conditions (such as with a high level of incrustation), as the cleaning has low water flow velocity requirements.

## 1. Introduction

Supplying consumers with high-quality drinking water is a challenging and complex process. It places high demands on water producers to ensure proper protection of water sources, its treatment, accumulation, and distribution to final consumers [1]. The total length of the drinking water network in Europe is 4,225,527 km [2]. This infrastructure requires maintenance and investments to provide clean and wholesome water for all. Therefore, some of the basic preconditions to ensure the supply of high-quality drinking water to all consumers are the proper operation of water infrastructure, its construction, renewal and maintenance, in particular [2].

The renewal of the water supply network and the development of water supply network monitoring significantly reduce water losses in the water supply network. In certain EU member states, the average daily demands (ADD) per capita are decreasing (e.g., in the Czech Republic ADD has practically halved since 1989 to date; from 1.25 million m^3^·y^−1^ to 585,000 m^3^.y^−1^) [3]. This has a positive effect especially in terms of saving water resources as the Earth is currently struggling with a decrease in the yield of underground resources [4] or flow in watercourses [5] in many localities. However, the water savings may have a negative impact on the water quality due to its longer residence time in the pipeline [6], [7], where the resulting water quality is altered in sensory and microbiological parameters (i.e., heterotrophic plate count) [8,9]. 

All EU countries place high demands on the quality of supplied drinking water, and water management companies have begun to value consumers as valuable customers [10], [11]. Therefore, efforts have been increasing to reduce the occurrence of adverse phenomena, such as turbid events in the water supply network, which the consumer feels the most. On the other hand, the distribution system can never be free from all particles or microorganisms, being a complex set of chemical and biological reactants [12]. However, the presence of sediments and microorganisms can be reduced significantly by a correctly selected and frequent maintenance method for their removal [13]. 

The commonly-used methods for distribution pipe cleaning [14] may be summarized as pigging, Ice Pigging^®^, air scouring (AS), and water flushing. Recently, Neutral Output Discharge Elimination System (NO-DES) method has been introduced, which functions as aboveground loops of distribution system between two hydrants using hoses and the water is circulated within the temporary loop at scouring velocities through the water main and filters using pump mounted on the truck [15]. The method most authors focus on is the so-called unidirectional flushing [12,16,17,18,19,20,21,22]. While the highest possible flow rates of water in the pipeline are achieved, accumulated sediment or parts of biofilm are removed from the pipe surface [19,21]. The principle of this method is summarized by [23] based on the knowledge of many authors. Recommended flow velocities are in the range of v = 1.0–1.6 m·s^−1^ [12,24]. These velocities are sufficient for the removal of loose and cohesive deposits [19] and partly for tuberculated pipes [24].

The water pipe can be clogged with fine sediment at the bottom of the pipe or by chemical deposits on the pipe walls, which can fill almost the entire pipe diameter [25]. These sediments may be soft, easily removable to solid requiring aggressive cleaning methods [7,18]. Therefore, a higher velocity and higher shear stress are necessary to clean the inner surface of the pipe and its adherent material [12,24]. However, this is often problematic when using the unidirectional flushing method in real operation conditions, which is confirmed by the results obtained. Higher shear stress can be achieved by air scouring and Ice Pigging^®^, each of these methods uses a different cleaning principle. During air scouring compressed air is blown into the pipe, together with a liquid, which increases the shear stress at the pipe walls and scours and flushes out the sediment from the pipe [12]. Air scouring has been modernized to a software-driven impulse flushing which is marketed under trade names. During Ice Pigging^®^ the friction at the pipe walls is 2–4 orders of magnitude higher than at the same speed of a water stream during flushing [26], because the friction is increased using two-phase ice slurry in form of a plug moving down the pipe and dislodging built-up material [27]. Mostly a mixture of 5% food salt and potable water is used in the process [28,29].

Information about the amount and origin of deposits is useful to prevent their formation [19], therefore authors evaluate the efficiency of removal and composition of the deposits. The majority of studies focus on unidirectional flushing [12,19,22,30]. However, it has been found that limited knowledge exists on the comparison of the modern methods, such as Ice Pigging^®^ and air scouring, even in terms of operational requirements or in terms of efficiency. The existing studies only compared unidirectional flushing or pigging [12,18] or examined their efficiency in laboratory conditions [31].

The aim of the research reported here is to provide novel information regarding the efficiency of Ice Pigging^®^ and air scouring based on loose and adherent deposits removal and total water consumption. The aim is to compare two commercial methods called Ice Pigging^®^ and software-driven impulse flushing Comprex^®^, and to contrast the results with these obtained by unidirectional flushing. This new knowledge on suitable operation conditions obtained by testing methods in practice can optimize the performance of the distribution system cleaning. Air scouring is not involved in general evaluation because only one application of AS was performed, and enough data was not obtained. Air scouring results are in the main text mentioned only for comparison with the effectivity of software-driven process Comprex^®^.

## 2. Methodology

### 2.1. Selected Network Characteristics

To address these objectives, full-scale tests were carried out in test zones in the water distribution system of a 300,000-citizen city, which consists of 1064.8 km of pipes with the capacity of 1.875 L·s^−1^. Almost 16.5 km of the distribution system was monitored during the cleaning processes applying Ice Pigging^®^, Comprex^®^ and unidirectional flushing. Ice Pigging^®^ was applied to 75% of investigated pipes length, unidirectional flushing to 13% of investigated pipes length, and Comprex^®^ to 12% of investigated pipes length. The average length of cleaned section was for (a) Ice Pigging^®^ l_IP_ = 1749.1 m; (b) Comprex^®^ l_CO_ = 363.3 m; (c) unidirectional flushing l_UNI_ = 197.3 m.

**Figure 1:**
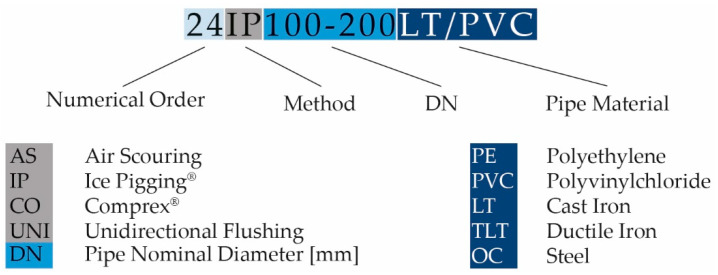
Code characterizing the experiments’ numerical order and cleaning conditions-used method, pipe nominal diameter and pipe material (in case of one cleaned section is created by more types, e.g. a hyphen (-) divides particular pipe nominal diameters and a slash (/) divides used types of pipe materials).

According to Macek and Škripko [16], it is possible to carry out unidirectional flushing up to pipe nominal diameter DN = 300 mm. Minimum recommended speed [12,24] for this nominal diameter is equal to the instantaneous flow of Q = 63.6 L·s^−1^. In the tested operating conditions, this flow is often limited by hydrodynamic pressure and hydrant capacity, therefore it was possible to flush effectively only sections up to DN = 150 mm. Comprex^®^ method is capable of cleaning pipes of nominal diameters up to DN = 1200 mm [32]. Candy et al. [33] state that Ice Pigging^®^ can be applied to clean the diameter up to DN = 750 mm. Sections up to DN = 450 mm have been tested under real operating conditions [33]. Based on this information, pipes suitable for cleaning were selected as follows (a) pipes from DN = 80 mm to DN = 150 mm in unidirectional flushing; (b) pipes from DN = 100 mm to DN = 250 mm in Ice Pigging^®^; (c) pipes from DN = 80 mm to DN = 200 mm in Comprex^®^ method. Tested localities were named by code (Figure 1), which characterizes the selected method and basic properties of the cleaned section, such as pipe diameter (DN) and pipe material. The frequency of the experiments was influenced by the cost of the methods being evaluated. No cleaning interventions have been reported since the pipe was commissioned. 

### 2.2. Fieldwork Procedure, Monitoring and Sampling

According to Vreeburg [12], Ellison [18], Carrière et al. [19], Barbeau et al. [22], Fann et al. [30] and Miller et al. [32], the effectiveness of pipe cleaning methods is often evaluated as total suspended solids removal. For this purpose, in-situ samples (volume of 2 L each) were collected and shipped to the laboratory for analysis. In the case of unidirectional flushing and Comprex^®^, sampling was carried out immediately after the start of cleaning. In the case of Ice Pigging^®^, the first sample was taken after a hydraulic test (described by Fann et al. [32]) and further samples at the moment as the front of the ice began to approach, which in most cases met one of the conditions: (a) Temperature dropped below 5 °C; (b) conductivity began to rise above normal levels in the area (up to Κ = 87.3 mS·cm^−1^).

Qualitative properties of flushed water (turbidity, temperature) were monitored on site during the whole process by digital optic sensor: (a) METTLER TOLEDO InPro 8000 Series (880 nm wavelength, Back-scattered Light principle, Mettler - Toledo, s.r.o., Praha, Czech Republic) for Ice Pigging^®^ and (b) PONSEL^®^ (850 nm wavelength; Side-scattered Light principle, TECHNOAQUA, s.r.o., Dolní Břežany, Czech Republic) for unidirectional flushing. In the case of Comprex^®^ method, turbidity was measured only by portable turbidimeter Eutech TN-100 (850 nm wavelength, Side-scattered Light principle, Fisher Scientific, spol. s r.o., Pardubice, Czech Republic), as the presence of air in the water impairs the accuracy of the continuous measurement. In all cases, the measurement of turbidity was complemented by measuring total iron by Ferrover^®^ method using Multiparameter Portable Colorimeter HACH DR900 (HACH LANGE s.r.o., Praha, Czech Republic). Other parameters measured in-situ are listed in Table 1 below as parameters measured on-site during the whole process using on-line sensors. 

### 2.3. Cleaning Parameters

Unidirectional Flushing was carried out at velocities ranging from 0.42 to 2.76 m·s^−1^ for metal pipes, and 0.73 to 1.87 m·s^−1^ for plastic pipes as a function of pipe diameter and available pressure. Only 4 out of 10 sections met the recommended speeds [12,24] for performing unidirectional flushing.

Air scouring without the possibility of airflow adjustment was performed only at one location as a test before Comprex^®^ cleaning. The used compressor was capable to create maximum pressure of 1 MPa and air injection was controlled only by front valve closing. Air pulses were created while the final water flow velocity was v = 0.28 m·s^−1^, while airflow could not be controlled.

Set operating parameters of Comprex^®^ cleaning method as a pressure of individual pulses ranged from 1.9 to 5.3 bar (190 kPa to 530 kPa), and the pulse length from 2 to 7 s. These parameters were a function of regular hydrostatic pressure in cleaned pipe and pipe material. A total of 179 to 537 pulses were used.

The volume of ice used for Ice Pigging^®^ is limited to 9 tons by ice manufacturing and storage capacity. This volume together with pipe diameter, material, flow rate and ambient temperature influence the total length of pipe which can be cleaned at a time [33]. The total volume of ice used for Ice Pigging^®^ cleaning varied 4–10 m^3^. Ice fraction varied from 75 to 85% (Mode of 80%).

The cleaning parameters, including the properties of the cleaned pipes, are summarized in Table 2. In cases where groundwater predominates over surface water (U > G) a specific groundwater-surface water ratio varies according to the instantaneous water-use and it cannot be accurately expressed. 

### 2.4. Determination of Total Water Consumption

As the water quality was monitored during the whole cleaning process, after reaching a turbidity value below 1 NTU, the mass concentration of total iron was measured. The cleaning was completed when total iron concentration met the requirements of Directive (EU) 2020/2184 of the European Parliament and of the Council of 16 December 2020 on the quality of water intended for human consumption. To reach the prescribed limits c(Fe) = 0.200 mg·L^−1^, the selected cleaning method had to be combined with conventional flushing.

The total water consumption was calculated as the total volume of water consumed from the start of the cleaning process to its end. The total consumption, therefore, included the volume of water consumed for the operation of the selected method and the volume of water consumed for conventional flushing until the drinking water quality standards were reached. The evaluation did not include possible loss in the event of a failure and its removal, if this failure occurred during cleaning.

### 2.5. Calculation of the Total Deposits Removed as TSS

The concentration of total suspended solids (TSS) in collected samples were determined using filtration through glass fiber filters according to European Standard EN 872:2005. Continuous monitoring of turbidity allowed to construct a curve of dependence of the immediate mass concentration of total suspended solids on turbidity and to calculate missing values to obtain more accurate results. The total amount of TSS in each experiment was calculated using the system for modern technical computing Wolfram Mathematica [34] as a sum of definite integrals of partial areas under a TSS concentration/consumed water volume curve. The values of the cumulative volume of water consumed for flushing were plotted on the *x*-axis and on the *y*-axis there were plotted the measured values of TSS corresponding to the consumed amount of water. A linear regression of the parts between the individual measurement points was performed and found equations were used to calculate the definite integral. The equations thus differed for each locality. 

### 2.6. Solid Phase Composition

The composition of solid phase was compared using Inductively coupled plasma optical emission spectrometry (ICP-OES) EN ISO 11885:2009, powder X-ray diffraction analysis (Bruker AXS D8 Advance 2Θ/Θ LynxEye, 40 KV/40 mA radiation, emission line Cu-Kalpha, Bruker EAS GmbH, Hanau, Germany) and electron microscopy using Quanta 650 FEG-Field Emission Scanning Electron Microscope (FEI Czech Republic s.r.o., Brno, Czech Republic) at low-vacuum-50 Pa, 1.4 nm @ 30 kV (SED). Organic matter in the solid phase of sediment was estimated from the loss on ignition (LOI_550_) of the mass of measured total suspended solids oxidized at 550 ± 5 °C; t = 1 h according to ČSN 75 7350:2008 Further on also as Volatile suspended solids (VSS). 

### 2.7. Liquid Phase Composition

Color, turbidity, metals and suspended solids concentration were analyzed in all the samples according to the methods of Inductively coupled plasma optical emission spectrometry (ICP-OES) EN ISO 11885:2009, gravimetric analysis EN 872:2005, spectrophotometry EN ISO 6271:2015 and nephelometry ASTM D7726-11(2016)E1.

## 3. Results and Discussion

Three calculation approaches were used to evaluate the amount of removed sediment in the form of TSS. The total amount of TSS from one specific section cleaning, which was determined by integration of the area under a TSS concentration/consumed water volume curve, was recalculated for a unit of length, a unit of volume and a unit of area of the cleaned pipe. All the results are summarized in Table 3 and divided according to implemented cleaning method and the chosen evaluation approach. As the results obtained from unidirectional flushing showed a dependence on the pipe material and sufficient data were obtained, this method of cleaning is in Table 3 divided into two sections UF of plastic pipes and UF of metal pipes. For each cleaning method, an average and a median are listed. The results are commented on in the following subsections. 

### 3.1. Total Sediments Removed as TSS

Total amount of removed impurities in the form of TSS determined by integration was recalculated per (a) unit length, (b) unit volume and (c) unit area of the cleaned pipeline. 

**Figure 2:**
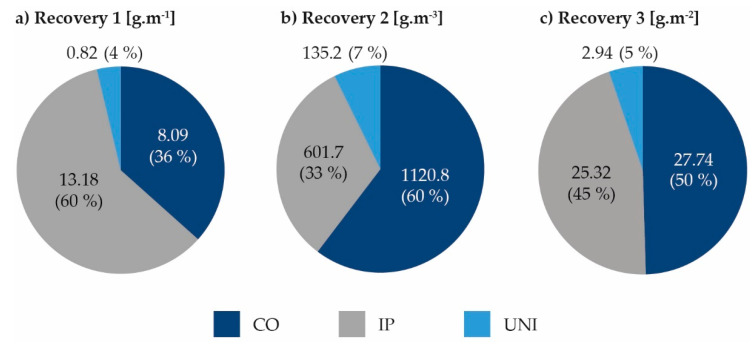
Effectiveness of methods depending on the chosen evaluation procedure per (**a**) unit length, (**b**) unit volume and (**c**) unit area of the cleaned pipe: The percentage was calculated as the ratio of the amount of TSS removed by the selected method to the total amount of TSS removed by three methods (IP−Ice Pigging^®^; UNI−Unidirectional flushing; CO−Comprex^®^).

The results differ depending on the chosen evaluation method. In case of (a) the most impurities were removed as follows: Ice Pigging^®^ (60%), Comprex^®^ (36%) and unidirectional flushing (4%). In case of (b) the most impurities were removed by Comprex^®^ (60%), Ice Pigging^®^ (33%) and unidirectional flushing (7%), and in case of (c) the most impurities were removed by Comprex^®^ (50%), Ice Pigging^®^ (45%) and unidirectional flushing (5%). The percentage was calculated as the ratio of the amount of TSS removed by the selected method to the total amount of TSS removed by all methods always related to a dimension unit. See also Figure 2. 

In the case of unit length (a) the smallest amount of removed TSS was gained during unidirectional flushing. Unidirectional flushing of plastic pipes removed 0.04–0.95 g·m^−1^ and unidirectional flushing of metal pipes removed 0.50–2.15 g·m^−1^. The total amount per unit length depended on the material of the cleaned pipe and its age, the parameters influencing hydraulic conditions. Comprex^®^ removed 1.63–17.40 g·m^−1^ (an average of 8.09 g·m^−1^ TSS of 5 applications) and Ice Pigging^®^ removed 1.77–25.27 g·m^−1^ (an average of 13.18 g·m^−1^ TSS of 7 applications). For comparison, Vreeburg [12], Carrière et al. [19] and Barbeau et al. [22] obtained by unidirectional flushing results of removed impurities in form of TSS from 0.1 to 0.4 g·m^−1^ and Miller et al. [35] using Ice Pigging^®^ from 0.1 to 58 g·m^−1^. However, comparisons based on unit length are possible to make only for pipes of identical nominal diameters. Therefore, this evaluation approach is not suitable in the case of different pipe nominal diameters and may distort the results. 

In case of unit volume (b) amount of removed TSS per unit volume, the least impurities were removed by unidirectional flushing of plastic pipes, namely 8.0–143.0 g·m^−3^ (61.0 g·m^−3^ of TSS in average) and unidirectional flushing of metal pipes 63.0–427.0 g·m^−3^ (209.4 g·m^−3^ of TSS in average). Comprex^®^ removed 208.0–3612.0 g·m^−3^ (1120.8 g·m^−3^ of TSS on average) and Ice Pigging^®^ removed 130.0–1065.0 g·m^−3^ (601.71 g·m^−3^ of TSS on average). Researchers do not use this expression of results very often, and no comparable data were found.

In the case of unit area (c), which is the most appropriate method of result expression as deposits can settle over the entire pipe surface [12]. The following results were achieved: unidirectional flushing of plastic pipes removed 0.12–3.01 g/m^2^ (an average of 1.37 g·m^−2^ TSS of 5 applications), 1.58–8.546 g·m^−2^ from the metal pipes (an average of 4.51 g·m^−2^ TSS of 5 applications). Comprex^®^ removed 5.19–69.23 g·m^−2^ (an average of 25.03 g·m^−2^ TSS of 5 applications), and Ice Pigging^®^ removed 4.36–47.53 g·m^−2^ (an average of 25.32 g·m^−2^ TSS of 7 applications). The amounts of sediment removed varied only in the multiples of tens to hundreds based on the chosen method. The 100–1000 times greater efficiency has not been achieved as is stated by Miller et al. [35]. Despite the fact, that the required flow rates in the pipeline were not reached in the case of unidirectional flushing of metal pipes, a larger amount of sediment per 1 m^2^ was removed from the metal pipes than from the plastic pipes. The larger amount of sediment per 1 m^2^ was also removed by Comprex^®^ method in metal pipes. The same observation was not valid for Ice Pigging method^®^, as there were often operational complications with the dosing of ice into the water supply system when cleaning metal pipes, which can have a significant effect on the cleaning process and total recovery of impurities. 

**Figure 3:**
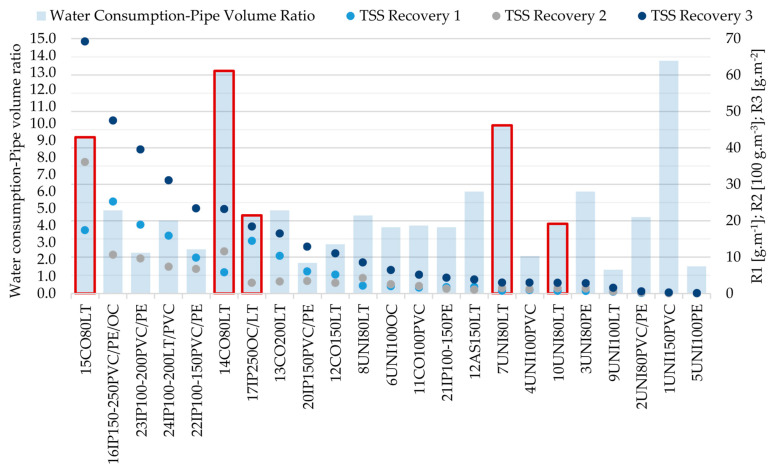
Total suspended solids and water consumption (red-framed columns indicate incrusted sections, for 18IP150-200OC/LT, 19IP80-150OC/LT/PE/PVC and 25IP150-200LT/PVC-data is not available).

Next, the results can be compared with the research reported by Fann et al. [30] who present the results of Ice Pigging^®^ and unidirectional flushing cleaning method of asbestos cement pipes DN = 150–400 mm, Miller et al. [35] who present the results for Ice Pigging of cast iron pipes DN = 200 mm, and Barbeau et al. [22] investigating unidirectional flushing of pipes of DN = 200 mm of cement-lined ductile iron and unlined grey cast iron. The results vary considerably: 2.91–28.85 g·m^−2^ for asbestos cement pipes, 90.498 g·m^−2^ and 23.13 g·m^−2^ for cast iron pipes, and 0.41 for grey cast iron and 0.64 g·m^−2^ for cement-lined ductile iron. The results may differ due to different samples’ volumes, sampling intervals or cleaning termination points. A commonly used limit for ending the cleaning is by achieving a turbidity value ≤ 5 NTU [17,19]. Different pipe age, which has a direct effect on the condition of the pipeline, can have a great effect (Figure 3). The comparison above confirms the findings of Barbeau et al. [22] that deposit accumulation (and its recovery) is highly site-specific.

To the authors’ knowledge, the results of the amounts removed as TSS are not available for the Comprex^®^ method or another method based on air scouring, so the values obtained cannot be compared.

### 3.2. Water Consumption

In terms of water consumption, cleaning was completed when the total iron concentration met the requirements of Directive (EU) 2020/2184, the evaluation of the methods was as follows: The Ice Pigging^®^ method consumed on average 3.5 times the volume of the cleaned pipe. The unidirectional flushing method consumed 5.19 times the volume of the cleaned pipe, and the Comprex^®^ method consumed 6.9 times the volume of the cleaned pipe. The water consumption of the Comprex^®^ method was greatly affected by sections with a high degree of incrustation, which consumed 9.2–25.3 times of the volume of the cleaned pipeline. The water consumption for unidirectional flushing of sections with an identical level of incrustation was similar (9.9–13.7 pipe volumes) as shown in Figure 3 (14CO80LT, 15CO80LT and 7UNI80LT), while removal of TSS was lower in the case of unidirectional flushing. It was not possible to clean such incrusted sections by Ice Pigging at all due to high friction and pressure loss (17IP250OC/LT and 18IP150-200OC/LT).

The obtained results comply with the data reported by Miller et al. [35], who reports half water consumption than in the case of unidirectional flushing, and Pourcel et al. [36], who reports the water consumption of 40% less in the case of air scouring than in the case of unidirectional flushing. This consumption only applies if the incrusted sections are not included in the evaluation. According to results by Fann et al. [30] the volume of water consumed in Ice Pigging^®^ was approx. 1.5 pipe volume. Authors in [37] identically state less than 2 pipe volumes for Ice Pigging^®^ and 4–7 for unidirectional flushing. Tan et al. [38] report a saving of 95% compared to using the unidirectional flushing when cleaning DN = 1400 mm pipes by air scouring. Differences can be caused by a number of factors, chosen monitored parameter (turbidity X total iron) and the value [15,39], when cleaning is completed. In the case of Ice pigging consumption may also be influenced if the total water consumption includes water consumed for the necessary hydraulic test. These data are not usually listed-see [30,37]. 

### 3.3. Maximum Impurities Concentration

**Figure 4:**
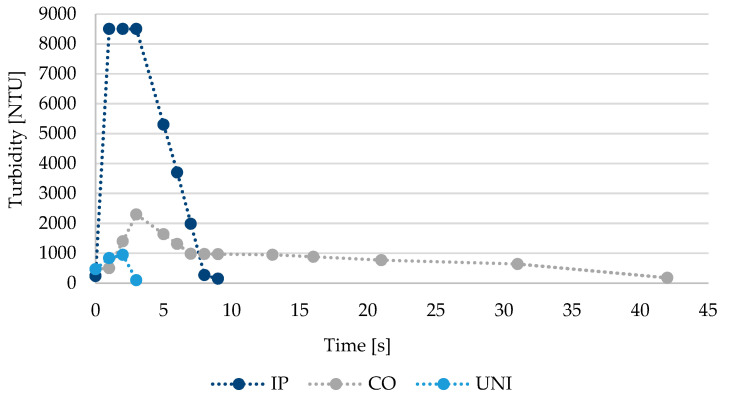
The distribution of the measured turbidity shows the principle by which particles are carried out of the pipeline (IP—Ice Pigging^®^, CO—Comprex^®^, UNI—Unidirectional flushing).

Regardless of the chosen cleaning method, there was always a sharp increase in measured values of observed chemical parameters (iron, manganese and aluminum mass concentration, color and turbidity) when the required hydraulic conditions (or the front of the ice) were reached. All the tested methods equally show a gradual decrease in the concentration of removed impurities until the moment when all parameters met the requirements of Directive (EU) 2020/2184 and the cleaning was finished. However, the time needed to remove the largest portion of impurities differed (Figure 4). Therefore, the achieved maximum values of TSS, color, turbidity and metals’ mass concentration also differ. The highest values of measured parameters were achieved using Ice Pigging^®^ when removed particles are absorbed the most in front of the ice method (TSS = 924–8100 mg·L^−1^; color > 4000 mg·L^−1^ Pt; turbidity > 7500 ZF(t); ρ(Fe) = 65.7–627 mg·L^−1^; ρ(Mn) = 3.06–329 mg·L^−1^; ρ (Al) = 32.1–274 mg·L^−1^). While in Comprex^®^ (TSS = 478–1200 mg·L^−1^; color = 480–2100 mg·L^−1^ Pt; turbidity = 66–2300 ZF(t); ρ(Fe) = 62.1–285 mg·L^−1^; ρ(Mn) = 1.06–67.7 mg·L^−1^; ρ(Al) = 1.15–22.9 mg·L^−1^) and unidirectional flushing (TSS = 6.4–450 mg·L^−1^; color = 190–1600 mg·L^−1^ Pt; turbidity = 35–950 ZF(t); ρ (Fe) = 0.573–138 mg·L^−1^; ρ(Mn) = 0.098–91.6 mg·L^−1^; ρ(Al) = 0.181–5.02 mg·L^−1^) particles are gradually entrained by the water stream, shear stress influences the results. Since samples taken during Comprex^®^ had a character of a mixed sample given by a single block of water, therefore the maximum measured concentrations of individual parameters probably do not correspond to the absolute maxima that were reached during cleaning and may be underestimated. The measured results thus showed that color, turbidity or concentration indicators are not suitable for comparing the methods between one other, as three tested methods showed a different distribution of measured values of color, turbidity and mass concentration of observed chemical parameters over time. No specific dependency was found between these indicators, because concentrations varied from one locality to another. Similarly, Pourcel et al. [40] tried to find a dependence between TSS and turbidity. Especially the metal content is strongly influenced by particle origin-the pipe material and its age, but may be affected also by the water source (which mainly affects the amorphous phase of the sediment as in Section 3.5). 

If plastic pipes made of polyethylene or polyvinylchloride were cleaned and hydraulic conditions were met in unidirectional flushing, the chosen sections were cleaned, and legislative limits were met very quickly. In the case of unidirectional flushing of metal pipes very often hydraulic conditions were not complied with, and it was always necessary to carry out conventional flushing to reach the legislative limits in total iron parameter. Otherwise, the fine iron particles remained suspended, legislation limits were still exceeded and pipe cleaning was unreasonably prolonged with a minimal cleaning effect. Very small particles bound to the pipe walls by weak electrostatic forces (Van der Waals’s forces) are likely released during unidirectional flushing [41].

Old metal pipes made of steel or cast iron cleaned by the Comprex^®^ method also showed a similar course. Iron particles were removed quickly, but the presence of particles of higher density prolonged the cleaning. These are probably released incrustations, which require higher shear stress to set sediment in motion [42]. Comprex^®^ method, therefore, releases larger particles than the unidirectional flushing method, which moves more slowly down the stream out of the pipe. Even in this case, it was appropriate to end the cleaning by conventional flushing, to re-settle the incrustations in the pipe and flush out any accumulated air. According to [43], the daily flow rate in the water supply system was found to be ≤ 0.12 m·s^−1^, therefore, it is advisable to flush at this flow velocity or lower. The flow velocity used in the given experiments corresponded to v = 0.066 m·s^−1^.

There were some limitations of sampling during Comprex^®^ because of the different nature of the movement of the sediment cloud through the pipeline caused by the alternation of air blocks and water blocks [18]. This principle causes (a) the formation of alternate stress during which particles are carried away, and (b) a change in the qualitative properties of water leaving within a single alternation. Therefore, every taken sample has the character of a composite sample of single impurities release and method principle probably affects the overall results, because the maximal measured concentrations of individual indicators (TSS, metal concentration, turbidity, etc.) probably do not correspond to the absolute maxima that were reached during the cleaning. Therefore, there is a probability that the effectiveness of Comprex^®^ method given in Figure 2 is even higher.

### 3.4. Hydraulic Capacity Assessment

Before Comprex^®^ cleaning of cast iron pipe of DN = 80 mm (14CO80LT) the maximum possible flow rate was measured as Q = 3.1 L·s^−1^. The same measurement was made after Comprex^®^ cleaning, but the maximum possible flow rate did not change and was on the same level. The hydraulic capacity of the cleaned pipes was not significantly improved. Probably, it means that only loose deposits were removed from the pipe. Cleaning will have a positive effect on water quality during peak demand. The average consumption is 0.038 L·s^−1^. 

### 3.5. Solid Phase Composition 

Solid-phase composition comparison was based on the ICP-OES method, powder X-ray diffraction and LOI_550_ analysis. For solid-phase composition comparison, only samples obtained during the Comprex^®^ method were analyzed (except LOI_550_ analysis), since the Comprex^®^ discharge passed the baffle box fitted with sieves where the sediment was captured and a sufficient amount of sample for powder X-ray diffraction analysis was obtained. Compared five sections varied in pipe material, year of commissioning or water source composition as it is given in Table 2. A density difference of solid-phase was investigated by an orientation test of measuring the sample volume at constant sample weight. At the same weight (m = 2 g) the volume was as follows: in 14CO80LT the volume was V = 2.4 mL, in 15CO80LT the volume was V = 7.15 mL, in 11CO100PVC the volume was V = 2 mL, in 12CO150LT the volume was V = 3.6 mL, and in 13CO200LT the volume was V = 2.8 mL. For this purpose, it is not suitable to compare localities with different water sources and pipe materials between one other. 

In addition to a different sediment density, a different elemental composition, was also recorded (Table 4). According to the powder X-ray diffraction analysis in the samples from the metal pipes, the most represented minerals were Fe minerals, which formed almost 90% of the crystalline form (in plastic pipes only 36%). The average concentrations of Fe in the produced water in the sources supplying the investigated areas range from 19.8 to 98 µg·L^−1^. Thus, the composition of the crystalline form of Fe minerals did not depend on the source of drinking water. The water source probably affected the composition of the amorphous phase of the sediment. A significant part of the iron contained in the sediment is probably the product of corrosion of the pipes through which the water is transported. Samples of metal pipes contained on average 80% α-FeOOH, 6% Fe_3_O_4_ and 3% γ-FeO(OH).

**Figure 5:**
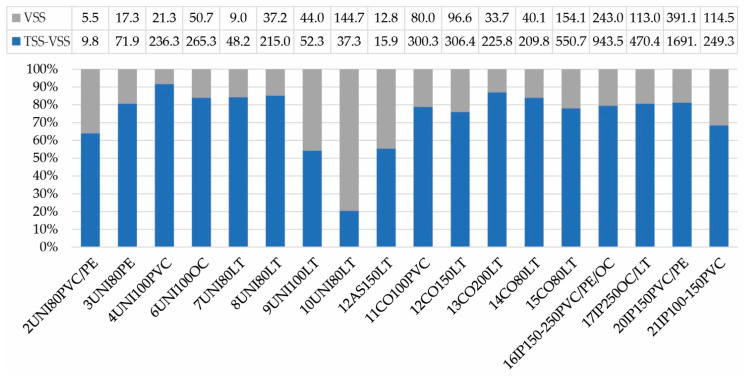
Ratio [%] of organic (VSS) and inorganic (TSS-VSS) material in solid phase of sediment estimated from the LOI_550_ of the mass of measured total suspended solids. More data are not available. VSS—Volatile Suspended Solids in mg·L^−1^; TSS-VSS—Residue on ignition in mg·L^−1^.

The measured values of Volatile suspended solids (obtained as LOI_550_) are listed in Figure 5. Unidirectional flushing samples contained an average of 22% organic matter, Comprex^®^ samples contained an average of 19% organic matter and Ice Pigging^®^ samples contained an average of 23% organic matter. The average organic matter of plastic and metal pipe samples was almost identical—22% in sediment from plastic pipes and 21% in the sediment of metal pipes. These results are in line with the research by Barbeau et al. [22]. The only difference was recorded in the evaluation based on the source of water supplying the studied drinking water system. Pipes supplied with a surface water source contained in sediment an average of 29% of organic matter, while pipes supplied with a mixture of groundwater and surface water or groundwater contained 18–19% of organic matter in sediments. This finding is in accordance with the fact that raw water from a surface source contains a higher proportion of organic substances than groundwater. Only the sediment picked from the cleaning 10UNI80LT contained almost 80% of the organic matter in the solid phase. In this section, the pipe was about 100 years old and probably the incrustation was excessive, because it was not possible to achieve the necessary hydraulic parameters and create sufficient shear stress to remove inorganic material of a higher density. Therefore, the proportion of organic and inorganic substances was probably different from the other cleaned sections, where the inorganic part formed the majority. According to WHO [13], deposits containing organic matter may support the growth of microorganisms and the formation of biofilms on internal surfaces of pipelines. Regular cleaning is, therefore, appropriate also from the viewpoint of preventing the suitable conditions for the development of pathogenic organisms, which can be attached to the biofilms. Due to the similar ratio of organic substances in all tested samples, the efficiency of organic matter removal depends on the efficiency of TSS removal (i.e., chosen method). 

Elements identified by the ICP-OES method can be adsorbed on the present organic compounds or on the previously mentioned iron oxohydroxides or manganese oxohydroxides MnOOH, the presence of which has been detected by electron microscopy in amorphous form in samples from 15CO80LT. Because a different sediment composition has a direct effect on the total weight of the TSS, which is removed from the pipe, therefore, a particle origin and a flushing method are the main variables affecting the total recovery. 

## 4. Conclusions

Since a suitable and correctly performed process of pipe cleaning of DWDS is one of the basic preconditions for ensuring the high quality of the supplied drinking water. Three commonly used methods of DWDS cleaning were selected for testing in real operation. These methods were Unidirectional Flushing, air scouring, Comprex^®^, and Ice Pigging.

We found during the experiments that all three tested methods are suitable for removing loose sediments, but their effectiveness differs based on the chosen form of evaluation (per unit length, unit area, or unit volume). Since contamination can settle along the entire surface of the pipeline, it is most appropriate to express the results per unit area of the pipeline. According to the obtained results, sediment in pipes differed in age, material and water sources and shows different properties (e.g., density), which directly affect the TSS result. It follows that evaluation of efficiency based on the amount of TSS removed is only suitable for sites that meet the same conditions (pipe material, water source and age ideally). The composition of the sediment was strongly influenced by particle origin, where the pipe material mainly affected the crystalline phase of the sediment and the water source and the age of the pipe affected the amorphous phase of the sediment.

It has further been found that the Comprex^®^ method can be advantageously used in real conditions to clean pipes with insufficient hydraulic conditions, as cleaning is not limited by the amount of cleaning medium. Areas with the maximum achievable flow rate Q_max_ = 3.3 L.s^−1^ (for DN 80 mm) were tested. This flow was determined for the use of the method and the given DN (at a lower flow no proper mixing of air and water in the pipeline occurred). According to the acquired experience, Ice Pigging^®^ is the most suitable for preventive maintenance of long straight pipe sections made of PVC, PE or metal pipes of cast iron, ductile iron and steel without advanced incrustation of DN 100–250 mm. This type of section was cleaned in a short period of time (7.9 m of pipes per minute), which can be advantageously used in the cleaning of sensitive areas with these parameters.

Although the overall recovery of the removed contamination is an important parameter for evaluating the effectiveness of the methods, for the above reasons it should not be the only decisive indicator. The advisable parameters are the measurement of relative clarity of drained liquid (as turbidity determined by attenuation of the radiant flux) or any concentration indicators chosen in dependence on the composition of the water sources supplying the area (including historical ones). These additional measurements can only be used to compare the cleaning results of one specific method due to the differences in the cleaning principles. 

Water consumption varied depending on the selected cleaning method. Within the studied drinking water system water consumption was the lowest using Ice Pigging^®^ and for unidirectional flushing and Comprex^®^, total water consumption was impacted by the condition of the cleaned pipe section. For non-incrusted sections, Comprex^®^ water consumption was lower than the water requirements of unidirectional flushing. If sections with high level of incrustation are cleaned the water consumption is similar. 

## Figures and Tables

**Table 1:** Parameters of water monitored on-site by on-line sensors.

Method	Ice Pigging^®^	AS & Comprex^®^	Unidirectional Flushing
Temperature	x		x
Pressure hydrostatic/hydrodynamic	x	x	x
Turbidity	x		x
Conductivity	x		
Flow rate	x	x	x

**Table 2:** Properties of the cleaned pipes and cleaning parameters (NDA—no data available; DN—nominal diameter; p—hydrodynamic pressure; QA—flow achieved; QR—flow required; WS—water source; G—groundwater— U—underground water).

Method	Code	DN	Commissioning	Material	Length	*p*	QA	QR	WS
		mm	Year		m	bar	L·s^−1^	L·s^−1^	
Unidirectional flushing	1UNI150PVC	150	1984	PVC	299.5	0.39	18.6	17.19	G
	2UNI80PVC/PE	80	2000–2007	PVC-PE	279.8	0.15	6.3	5.41	G
	3UNI80PE	80	2001	PE	122.9	0.28	9.4	4.30	G + U
	4UNI100PVC	100	NDA	PVC	199.2	0.41	6.6	8.01	U > G
	5UNI100PE	100	2015	PE	134.2	0.05	5.8	6.36	U > G
	6UNI100OC	100	1964	OC	93.5	0.06	4.5	7.85	U > G
	7UNI80LT	80	1977	LT	135.9	0.04	3.1	5.02	U
	8UNI80LT	80	1988	LT	250.5	0.28	13.9	5.02	G + U
	9UNI100LT	100	1971	LT	314.2	0.07	3.8	7.85	U > G
	10UNI80LT	80	1920	LT	143.5	0.07	2.1	5.02	U > G
AS	12AS150LT	150	1985	LT	198.8	NDA	5.0	-	G
Comprex^®^	11CO100PVC	100	1975	PVC	371.0	0.50	2.1	-	G + U
	12CO150LT	150	1985	LT	654.3	1.00	2.7	-	G
	13CO200LT	200	1970	LT	481.7	0.70	6.9	-	U
	14CO80LT	80	1977	LT	135.9	0.50	1.4	-	U
	15CO80LT	80	1953	LT	173.5	1.50	0.9	-	G
Ice Pigging^®^	16IP150-250PVC/PE/OC	150, 250	1964–2001	PVC-PE-OC	1661.0	5.50	15.2	3.00	U
	17IP250OC/LT	250	1964	OC-LT	1381.0	5.80	13.8	3.00	U
	18IP150-200OC/LT	150, 200	1953–1966	OC-LT	633.0	NDA		3.00	G
	19IP80-150OC/LT/PE/PVC	80, 150	1953–1994	OC-LT-PE-PVC	1005.0	NDA		3.00	G
	20IP150PVC/PE	150	1994–2008	PVC-PE	2052.0	3.80	14.2	3.00	G
	21IP100-150PE	100, 150	2009–2012	PE	2327.0	2.80	11.5	3.00	G
	22IP100-150PVC/PE	100, 150	1987–2016	PVC-PE	2638.0	0.50	8.8	3.00	U
	23IP100-200PVC/PE	100, 200	1997–2011	PVC-PE	1495.0	0.60	7.5	3.00	G
	24IP100-200LT/PVC	100, 200	1979	LT-PVC	687.0	0.40	14.5	3.00	G
	25IP150-200LT/PVC	150, 200	1979	LT-PVC	323.0	NDA	14.5	3.00	G

**Table 3:** Summary of total sediments recovery as TSS per unit length, unit volume and unit area and water consumption of tested methods including average and median. (UNI—Unidirectional flushing; AS—Air scouring; Recovery 1—Total TSS calculated per length of the pipe; Recovery 2—Total TSS calculated per the inner volume of the pipe; Recovery 3—Total TSS calculated per the inner area of the pipe; * estimated water consumption).

Method	Code	Recovery 1	Recovery 2	Recovery 3	Water Consumption-Pipe Volume Ratio
		g·m^−1^	g·m^−3^	g·m^−2^	
UNI of plastic pipes	1UNI150PVC	0.14	8.0	0.29	13.7
	2UNI80PVC/PE	0.15	29.0	0.59	4.5
	3UNI80PE	0.72	143.0	2.86	6.0
	4UNI100PVC	0.95	120.0	3.01	2.2
	5UNI100PE	0.04	5.0	0.12	1.6
	Average	0.40	61.0	1.37	5.6
	Median	0.15	29.0	0.59	4.5
UNI of metal pipes	6UNI100OC	2.03	258.0	6.46	3.9
	7UNI80LT	0.76	151.0	3.01	9.9
	8UNI80LT	2.15	427.0	8.54	4.6
	9UNI100LT	0.50	63.0	1.58	1.4
	10UNI80LT	0.74	148.0	2.96	4.1
	Average	1.23	209.4	4.51	4.78
	Median	0.76	151.0	3.01	4.10
AS	12AS150LT	1.82	103.0	3.86	6.0
Comprex^®^	11CO100PVC	1.63	208.0	5.19	4.0
	12CO150LT	5.20	294.0	11.04	2.9
	13CO200LT	10.37	330.0	16.50	4.9
	14CO80LT	5.83	1160.0	23.21–50.31	13.1–25.3 *
	15CO80LT	17.40	3612.0	69.23	9.2
	Average	8.09	1120.8	27.70	6.9
	Median	5.83	330.0	16.50	4.9
Ice Pigging^®^	16IP150-250PVC/PE/OC	25.27	1065.0	47.53	4.9
	17IP250OC/LT	14.46	295.0	18.41	4.6
	18IP150-200OC/LT	No data available			2.5
	19IP80-150OC/LT/PE/PVC	No data available			2.2
	20IP150PVC/PE	6.07	344.0	12.89	1.8
	21IP100-150PE	1.77	130.0	4.36	3.9
	22IP100-150PVC/PE	9.86	677.0	23.39	2.6
	23IP100-200PVC/PE	18.91	965.0	39.58	2.4
	24IP100-200LT/PVC	15.90	736.0	31.10	4.3
	25IP150-200LT/PVC	No data available			3.1
	Average	13.18	601.7	25.32	3.2
	Median	14.46	677.0	23.39	2.6

**Table 4:** Overview of measured element concentrations in solid phase by ICP-OES.

Element	Ca	Mg	P	Al	Cr	Mn	Cu	Ni	Pb	Na	Zn	Fe	Be
Code	mg·g^−1^												
11CO100PVC	10.3	2.02	0.77	9.75	0.281	17.50	0.575	0.390	0.516	1.200	43.9	145	0.001
12CO150LT	19.9	1.75	0.60	6.74	0.051	5.12	0.037	0.081	0.009	0.417	0.4	181	0.000
13CO200LT	17.5	1.43	1.79	5.62	0.215	4.00	0.223	0.205	0.041	0.336	1.3	505	0.000
14CO80LT	11.3	2.73	2.41	5.51	0.201	3.37	0.826	0.198	0.896	0.474	81.5	346	0.001
15CO80LT	22.4	3.43	1.22	34.00	0.038	148.00	0.312	1.580	0.133	0.611	8.4	251	0.001

## Data Availability

Data is contained within the article.
